# Microcomputer control and data system for automated multiple flow injection analysis

**DOI:** 10.1155/S1463924681000515

**Published:** 1981

**Authors:** James F. Brown, Kent K. Stewart, Darla Higgs

**Affiliations:** 1 Nutrient Composition Laboratory Beltsville Human Nutrition Research Center, Human Nutrition Science and Education Administration US Department of Agriculture Beltsville Maryland 20705 USA; 2 Nexus Associates 6500 Hanover Heights Trail Clifton VA 22024 USA


### Microcomputer control and data system for automated multiple flow injection analysis*

**James F. Brown**, Kent K. Stewart and Darla Higgs**

*Nutrient Composition Laboratory, Beltsville Human Nutrition Research Center, Human Nutrition, Science and Education Administration, US Department of Agriculture, Beltsville, Maryland 20705*

### Introduction

Flow injection analysis has been shown to be a versatile technique for the analyses of many types of samples using unsegmented continuously flowing streams. Several recent reviews have discussed a variety of aspects of this field [1-5]. The automated version of flow injection analysis (usually called automated multiple flow injection analysis or AMFIA) is attractive for those laboratories processing large numbers of samples or which require good precision, and several workers have been exploring this type of flow injection analysis [6-25]. There are three general types of AMFIA systems (Figure 1): the standard configuration with low or medium dispersion, the titration system with the large dispersion and the dilution system with low dispersion [22]. Each type of AMFIA configuration requires precise control of the sample tray, probe and sample injection valve. The dilution system also requires the operation of a fraction collector. Data must be acquired and processed by both the standard and titration systems. In this communication methods are described for the automation of all three types of AMFIA systems. The system described uses the Rockwell AIM-65 microcomputer, some associated electronics and layered user-oriented software. This work is a continuation of earlier efforts to provide full automation of AMFIA systems [22,23]. Malmstadt *et al* and Slanina *et al* have also described the use of computers for the control of FIA systems [19,24,25].

### Instrumentation

A Rockwell AIM 65 was selected to provide the control and data functions required by the AMFIA systems. The AIM 65 has several attractive features, eg low cost, terminal-style keyboard, 20 column printer and display, and 8K ROM (read only memory) BASIC compiler. The AIM 65 is based on the 6502 microprocessor. Interfaces are provided on the board for two cassette recorders using continuous or block mode recording techniques for 110-9600 BAUD serial communication and for 20 programmable I/O (input/output) lines. The monitor firmware (8K) also includes an assembler, text editor and many useful subroutines.

Figure 2 shows a block diagram of the interconnections between the AIM 65 and AMFIA hardware. The sampler and sample injection valve are controlled directly by the AIM 65 through optically isolated solid-state relays. The sampler probe has "SAMPLE" and "INJECT" states which are activated by a continuous voltage (110 AC) at either of two locations on a microswitch that is mechanically linked to the sampler motor. Transition from the "SAMPLE" to "INJECT" state automatically advances the sample tray. The pneumatic sample injection valve is activated by a 4-way solenoid valve and is configured in the "INJECT" position when the AC power is off; this arrangement reduces the level of electromagnetic interference (EMI) during data acquisition by the computer.

The fraction collector tray advance mechanism is activated through its drop counting circuit. During normal operation, the fraction collector can be preset to change collection tubes after 1 to 990 drops. To allow computer control, a 3-way manual switch was placed in the counter circuit (Figure 2) to route the current, normally through the drop sensor (a photo diode), to an optically isolated solid-state relay. The AIM 65 simulates the drops by breaking closure in the drop-counting circuit. A low-pass filter in the control limits false triggering due to noise.

As shown in Figure 2, filtered detector signals are amplified by a low-impedence ($10^{-13}$ ohm) operational amplifier and digitised by a 12-bit analog-to-digital converter. A wide range of detector voltages (between -20 and +20 volts) can be evaluated by adjusting potentiometers and changing jumpers connected to the integrated circuit amplifier and A/D converter. The 12-bit analog-to-digital conversion yields a resolution of one part in 4096 or 1/100 mV for a typical 50 mV full scale signal. Detailed schematics and a parts list are available upon request from the authors.

### Software

The instrument control and data system (ICDS) was developed to provide three levels of operation depending on the operator, analysis and hardware configuration. When one of the typical AMFIA configurations is used to perform a well-defined routine analysis, the system can be operated as a "turnkey" instrument by persons with minimal training. At a higher level of user sophistication with typical AMFIA hardware, the turnkey can be modified to perform novel analytical procedures. Operating the system in the turnkey mode and programming the functions to be carried out during turnkey operation are both supervised by the ICDS program. Expansion of the system to include different hardware and software must be performed outside ICDS program control and requires a moderate knowledge of electronics and of programming in BASIC and 6502 assembly language. Depending on the needs and training of the operator, predetermined operations may be executed in the turnkey mode, a new set of "turnkey" operations may be set up in the program mode or the ICDS operating system may itself be modified. Complete flexibility is available to the analyst in setting up the turnkey, while the demands on the operator in executing the turnkey are very simple.

* *A preliminary report of this work was presented by J.F. Brown and K.K. Stewart at the 31st Pittsburgh Conference on Analytical Chemistry and Applied Spectroscopy, March 10-14, 1980, paper number 83.*

** *Present Address: Nexus Associates, 6500 Hanover Heights Trail, Clifton, VA 22024, USA.*

The ICDS software and data occupy three regions of the AIM 65 memory, and each can be saved or retrieved independently. Thus, new programs or data can be loaded in one region without disturbing others. An application of this capability would be the insertion of extensive BASIC calculation programs to operate on blocks of old data that had been stored on cassette tape. The first section of the AIM 65 memory is allocated to the BASIC program. The latter regions are allocated to the program/data stack and to the assembly language programs. The BASIC program interacts with the user, supervises execution of user programs and performs calculations. Since BASIC programs execute relatively slowly, tasks such as real-time peak measurements are programmed in assembly language. Sample data and user programs are stored in a 130 by 3-byte stack outside the BASIC memory region to permit packing as hexadecimal numbers. This conserves memory and also provides file protection. User programs are stored from the top down and sample data is stored from the bottom up. The stack holds information for N samples and 130-N program lines. In practice most user programs are less than 10 lines long and therefore storage is generally available for up to 120 samples in one or more runs.

Six user program instructions provide three timed output states, a conditional jump and two data acquisition modes. As described in Table 1, each instruction has one or more arguments that are specifically called by prompts display as the instruction is entered into the user program. Each instruction performs a complete functional task required by the AMFIA system. The timed output instructions specify the states of four relays for periods up to 100 minutes in 1/10 second increments. As their names imply, "SAMPLE" fills the sample loop, "INJECT" places the sample in the stream to the detector and "PULSE 4" briefly interrupts relay 4 to advance the fraction collector or to activate an event marker on a recorder. Other relay configurations may

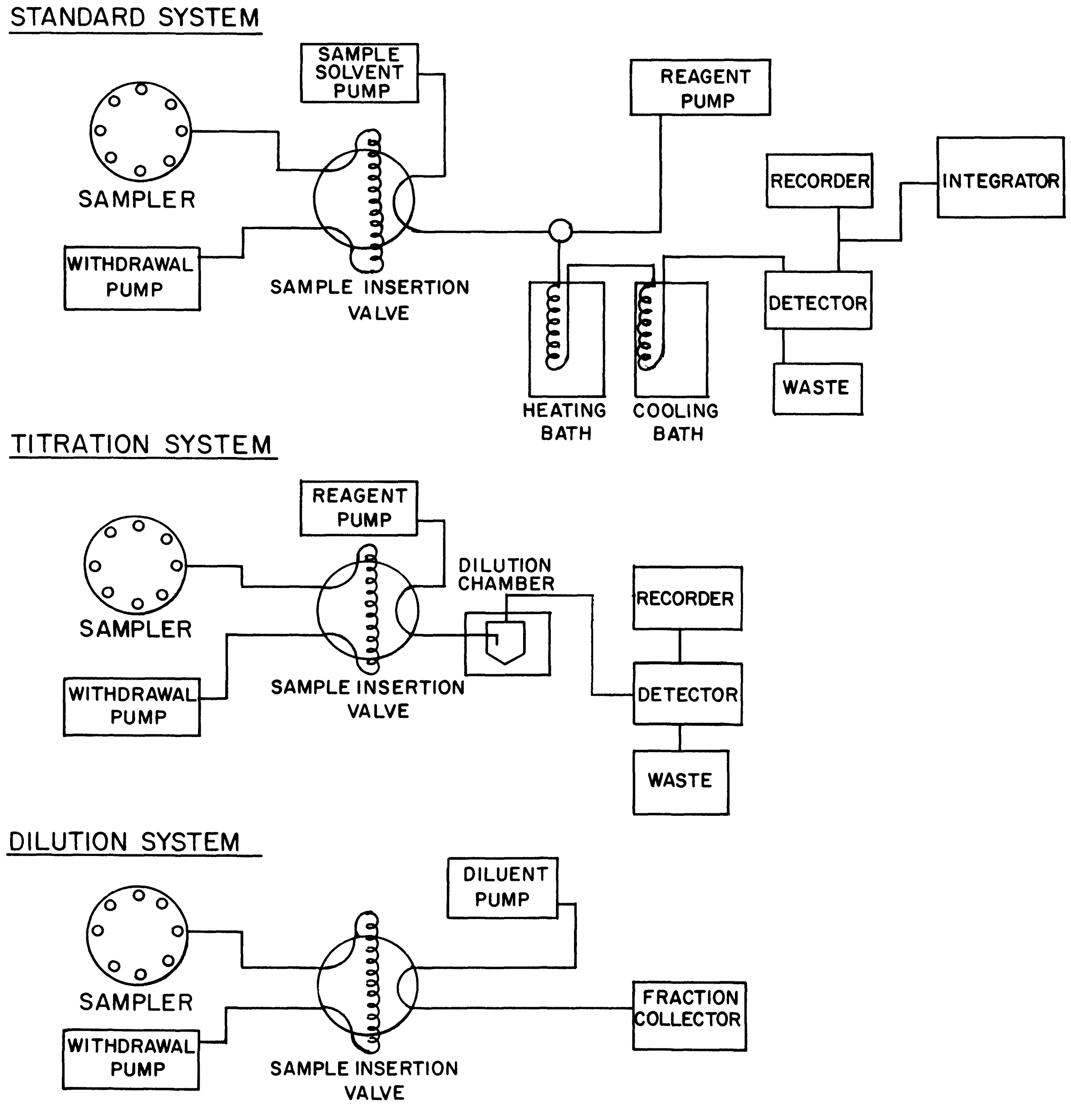

*Figure 1. Three AMFIA systems*

be easily defined to specify up to 16 timed output states, and the time increments are programmable.

The "JUMPIF" instruction will cause conditional or unconditional jumps to any user program line or stop the program. The first argument for this instruction specifies the input condition from the A/D converter under which the jump is to occur. If the input equals or exceeds the argument value, then program execution jumps to the program line specified in the second argument. Since the A/D output to the AIM 65 is always at least zero, unconditional jumps can be programmed by setting the first argument to zero. A value assigned to the second argument that is beyond available user program space will terminate execution of the user program.

Sample peak area is an essential measurement for standard AMFIA operations, and peak width is required for titrations. Acquisition of data for sample peak area or width is accomplished with the "GETPKA" or "GETPKW" instructions respectively. Both measurements are taken at programmable rates between 20 and 1000 Hz and may have values of up to about 16 million ($2^{24}$). Peak areas are integrated inside a time window of up to 65536 counts. Peak width measurements used for AMFIA titrations are begun after the detector signal persists above a threshold that is set by the first argument of the GETPKW instruction and ended when the detector output persists below the value of the second argument. The second argument must always be less than the first argument. At the conclusion of each area or width evaluation, the cup number and sample data are printed and stored in memory. The pre-integration baseline is also printed for data corrections and identification of system failures.

Printed copies of these programs are available upon request from the authors.

### Operation

A flow diagram of the display prompts, the operator keyboard entries and the printer outputs is shown in Figure 3 for the turnkey mode. The turnkey operation starts by printing the assay name and prompting entry of up to 60 characters of run identification. When the run identification has been entered, the assay name, identification, standard concentrations and user program are printed as part of the experiment record, and the display prompts the user to enter the first cup number and the number of cups in the run. These values are then printed and the display queries whether or not to execute the analysis. A negative response at this point returns the program to the beginning request for "RUN ID", and an affirmative response initiates the analysis. As the run is executed, the active program function and number of counts to the next program step are displayed. When an

**Table 1. The six words of ICDS and their associated arguments and numerical ranges**

| Command | Argument(s) | Range(s) |
|---|---|---|
| SAMPLE | Time | 1-65536 |
| INJECT | " | " |
| PULSE4 | " | " |
| JUMPIF | Condition/line | 0-255/1-130 |
| GETPKA | Rate/time | 0-255/1-6553 |
| GETPKW | Rate/level up/down | 0-255/0-255/0-255 |

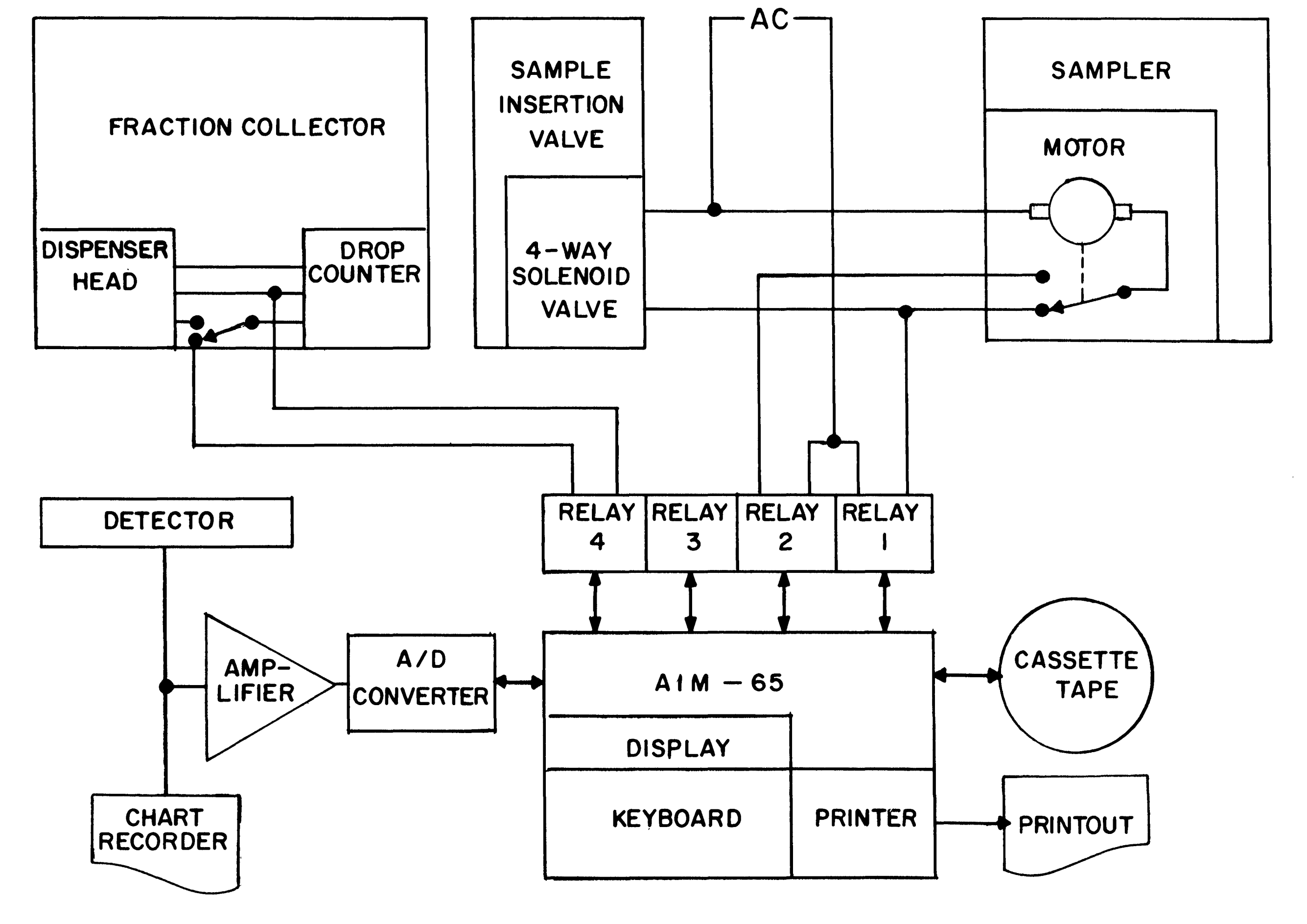

*Figure 2. Block diagram of the AMFIA – AIM-65 interconnections*

individual analysis is complete, the cup number, peak area/ width and base-line are printed. At any time during the run, program execution can be interrupted for hardware adjustments by hitting the F1 special function key and then continued from the same point or with any cup number by entering "CONT". When the analysis of all samples is complete (which may include multiple runs), a standard curve is calculated using a least squares fit of the standard concentrations and data from the first and last six samples in the data set. The printout includes the assay name, "RUN ID", standards, user program, area or width, and concentration. Data can also be stored on cassette tape or transmitted serially to a central computer system (programs for communication between the AIM 65 and other computers will be presented elsewhere). During turnkey operation, the operator need only confirm the assay name, supply identificaton of the samples in the run and specify the number of cups and starting location.

If it becomes necessary to generate a new turnkey system, the analyst can enter the edit mode by typing "EDIT" in response to the "RUN ID" prompt (Figure 3). In the edit mode, the analyst can list the area/width data or the current program, alter the existing turnkey program and standard values or return to the turnkey mode. Individual parameters can be altered without disturbing the rest of the system. The six program words (see Table 1) available for control and data processing in ICDS are sufficient for the typical AMFIA operations in the author's laboratory; most assays can be performed with four-line programs. Figure 4 shows a program for operating a standard AMFIA system (output and data aquisition arguments are in tenths of second). Line 1 of the user program causes the sampler probe to dip into the sample and the sample injection valve to switch to the load position for 15 seconds (150 tenths). The "INJECT" instruction on line 2 removes the sample probe from the sample cup, advances the sample tray and switches the sample injection valve to the inject position. When 3 seconds has elapsed, the "GETPKA" instruction integrates the detector signal for 35 seconds. The "JUMPIF" instruction on line 4 then returns control to line 1 if further samples remain to be analyzed. For titrations, line 3 might read "GETPKW 50 25" with a starting and terminating thresholds of 50 and 25, respectively (full scale is 4095). Dilutions would be implemented by replacing line 3 with "PULSE 4 1" to advance the fraction collector.

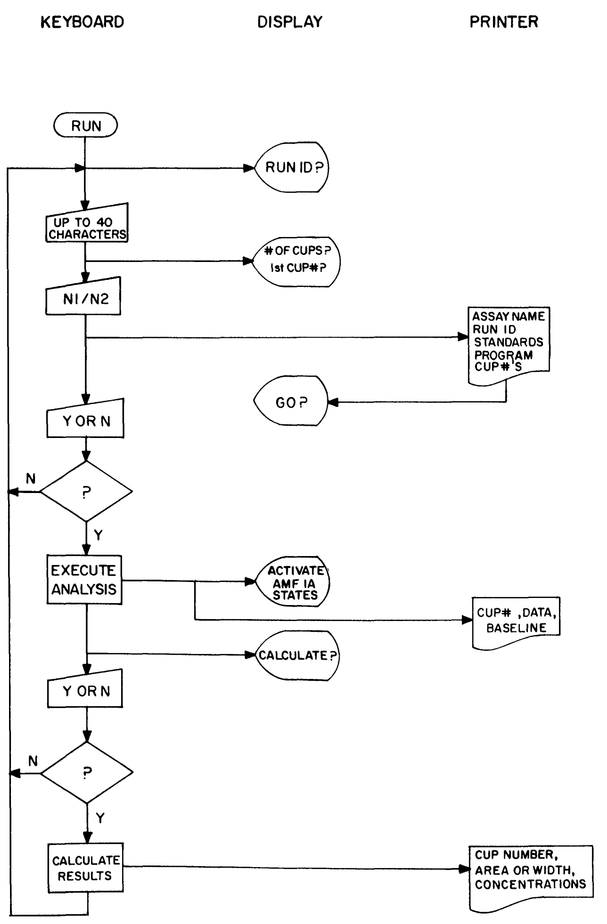

*Figure 3. Flow diagram for the AMFI turnkey operations*

When radical changes in the system are required, such as modifying integration techniques or the addition of more input/output lines to the AIM 65, the sophisticated user is not limited by ICDS software. The AIM 65 programming languages (assembly, PL 65, BASIC, FORTH, etc) can be used to expand the properties of ICDS or build entirely new operating systems.

### Discussion

Routine chemical analyses of large numbers of samples are required by many divergent professions in such fields as medicine, public health, agriculture and industry. The developer of automated analytical systems is challenged to accommodate the range of professional training and levels of computer skills found in these environments. Analytical protocols are often well defined for routine analysis, and microprocessor controlled systems designed to automate them can function satisfactorily with little user input. These turnkey instruments are attractive since they can be operated after minimal training, require no prior experience with computers, are insulated from user carelessness or ignorance, yet can operate complex assay systems. However, these same systems must often perform concurrently in a research environment where experimental conditions and methods are less well established. Here turnkey instruments often do not allow the sophisticated user adequate control and computational flexibility, and a less rigid system would be desirable. A common response to this conflict is to abandon automated equipment in the research setting or to purchase a different instrument that requires considerable user sophistication. The two-instrument approach is expensive and is a barrier to the transfer of methodology from the research environment to the routine analysis laboratory.

The systematic development of automated analytical instruments that serve both expert and novice is important for the continuing development and acceptance of automated chemistry. Microprocessor controlled instruments should allow not only turnkey operation but structured alteration of all functional parameters and full access to the microprocessor and its associated peripherals. The modular, inexpensive control and data system for automated multiple flow injection analysis described in this communication is an attempt to provide this type of instrumentation. The ICDS software was developed to simplify the demands on the user in operating the various configurations of AMFIA without compromising the potential for system expansion and change. In the simplest case, the user need only load the ICDS software into the AIM 65 from a cassette tape by following a few monitor

| Program line | Command | Argument(s) |
|---|---|---|
| 1 | SAMPLE | 150 |
| 2 | INJECT | 30 |
| 3 | GETPKA | 350 |
| 4 | JUMPIF | 0 1 |

*Figure 4. A typical ICDS program for the control of a standard AMFIA*

display prompts, enter the run identification and sample cup locations, and request calculations at the end of the analysis. The final printout supplies hard copy of the experiment, and the data can be saved on cassette tape through a simple prompted procedure.

In circumstances requiring greater control and computtational flexibility, the system allows limited (ICDS) or complete access to the microcomputer hardware and software and illustrates a stratified and alterable approach to automated systems software development. At one level the analyst can change the turnkey program and parameters essential to AMFIA operation without having to know the AIM 65 programming languages; at another level, the entire system can be changed. In the author's laboratory it has often been desirable for an "intelligent" instrument to control some associated but originally unrelated device or to include new features in its program. The system described here provides the potential for changes such as these that were not envisaged by designers at the time of instrument development.

The concepts discussed in this communication are a further extension of the basic premises that automated instrumentation should have modular hardware and software and should be useful at several levels of sophistication. The rapid proliferation of microprocessors in the laboratory, the evolution of user sophistication with micro-electronic devices and resurgence of the concept of modularity make it appropriate to implement these features whenever possible.

ACKNOWLEDGEMENTS
This work was supported in part by an interagency reimbursable agreement no 2Y01 HB60041 05 from the National Heart, Lung, and Blood Institute, NIH.

DISCLAIMER
Mention of trademark or proprietary products do not constitute a guarantee or warranty of the product by the US Department of Agriculture and does not imply their approval to the exclusion of other products that may also be suitable. It is the policy of the USDA not to endorse those commercial products used in the research over those not included in the research.

REFERENCES
[1] Betteridge, D. (1978), *Anal Chem,* **50**, 832A-846A.
[2] Ruzicka, J. and Hansen, E.H. (1979), National Bureau of Standards Special Publication 519, Trace Organic Analysis: A New Frontier in Analytical Chemistry, Proceedings of the 9th Materials Research Symposium, April 10-13, 1978, held at NBS, Caithersburg, MD, Editors H.S. Hertz and S.N. Chesler, 501-507.
[3] Ruzicka, J. and Hansen, E.H. (1980), *Anal Chim Acta,* **114**, 19-44.
[4] Ranger, C.B. (1981), *Anal Chem,* **53**, 20A-32A.
[5] Stewart, K.K. Talanta, in press.
[6] Stewart, K.K., Beecher, G.R. and Hare, P.E. (1974), *Federation Proceedings,* **33**, 1439.
[7] Beecher, G.R., Stewart, K.K. and Hare P.E. (1975), Protein Nutritional Quality of Foods and Feeds, Part 1, Proceedings of a symposium entitled "Symposium on Chemical and Biological Methods for Protein Quality Determination" Sponsored by the Agricultural and Food Chemistry Division of the American Chemical Society. 168th American Chemical Society National Meeting, Atlantic City, NJ, September 1974. E.M. Friedman, Marcel Dekker, Inc., New York, NY, 411-421.
[8] Stewart, K.K., Beecher, G.R. and Hare, P.E. (1976), *Anal Biochem,* **70**, 167-173.
[9] Beecher, G.R. and Stewart, K.K. Tenth Int Cong Biochem (1976), Abstract No 13-1-199.
[10] Stewart, K.K., Beecher, G.R. and Hare, P.E. (1977), US Patent 4,013,413
[11] Basson, W.D. (1977), *Lab Pract,* **26**, 541-545.
[12] Basson, W.D. and Van Staden, J.F. (1978), *Analyst,* **103**, 296-299.
[13] Basson, W.D. and Van Staden, J.F. (1978), *Analyst,* **103**, 998-1001.
[14] Basson, W.D. and Van Staden, J.F. (1978), *Lab Pract,* **27**, 863-865.
[15] Baadenhuijsen, H. and Seuren-Jacobs, H.E.H. (1979), *Clin Chem,* **25**, 443-445.
[16] Wolf, W.R. and Stewart, K.K. (1979), *Anal Chem,* **51**, 1201-1205.
[17] Basson, W.D. and Van Staden, J.F. (1979), *Analyst,* **104**, 419-424.
[18] Kawase, J. (1980), *Anal Chem,* **52**, 2124-2127.
[19] Malmstadt, J.V., Walczak, K.M. and Koupparis, M.A. (1980), *Amer Lab,* September, 17-40.
[20] Renoe, B.W., Stewart, K.K., Beecher, G.R., Wills, M.R. and Savory, J. (1980), *Clin Chem,* **26**, 331-334.
[21] Shideler, C.E., Stewart, K.K., Crump, J., Wills, M.R., Savory, J. and Renoe, B.W. (1980), *Clin Chem,* **26**, 1454-1458.
[22] Stewart, K.K., Brown, J.F. and Golden, B.M. (1980), *Anal Chim Acta,* **114**, 119-127.
[23] Stewart, K.K. and Rosenfeld, A.G. (1981), *J Automatic Chemistry,* **3**, 30-32.
[24] Slanina, J., Bakker, F., Bruyn-Hes, A. and Mols, J.J. (1980), *Anal Chim Acta,* **113**, 331-342.
[25] Slanina, J., Lingerak, W.A., Bakker, F. (1980), *Anal Chim Acta,* **117**, 91-98.

